# Direct observation of neighborhood attributes in an urban area of the US south: characterizing the social context of pregnancy

**DOI:** 10.1186/1476-072X-5-11

**Published:** 2006-03-17

**Authors:** Barbara A Laraia, Lynne Messer, Jay S Kaufman, Nancy Dole, Margaret Caughy, Patricia O'Campo, David A Savitz

**Affiliations:** 1 Department of Nutrition and Carolina Population Center, CB# 8120, University of North Carolina, Chapel Hill, NC 27599, USA; 2 Department of Environmental Sciences and Engineering, University of North Carolina, and National Health and Environmental Exposures Research Laboratory, US Environmental Protection Agency Human Studies Division, Chapel Hill, NC 27599, USA; 3 Department of Epidemiology and Carolina Population Center, CB# 7435, University of North Carolina, Chapel Hill, NC 27599, USA; 4 Carolina Population Center, CB# 8120, University of North Carolina, Chapel Hill, NC, 27599, USA; 5 School of Public Health, 5323 Harry Hines Blvd, V8.112A, University of Texas, Dallas, TX, 75390-9128, USA; 6 Centre for Research on Inner City Health, St. Michael's Hospital; Department of Public Health Sciences, University of Toronto, Canada; 7 Center of Excellence in Epidemiology, Biostatistics, and Disease Prevention, Mount Sinai School of Medicine, One Gustave L. Levy Place, Box 1057, New York, New York, USA

## Abstract

**Background:**

Neighborhood characteristics have been associated with poor maternal and child health outcomes, yet conceptualization of potential mechanisms is still needed. Census data have long served as proxies for area level socioeconomic influences. Unique information captured by neighborhood inventories, mostly conducted in northern US and Canadian urban areas, has shown important aspects of the community environment that are not captured by the socioeconomic and demographic aggregated individual statistics of census data. In this paper, we describe a neighborhood data collection effort tailored to a southern urban area.

**Methods:**

This study used data from the Pregnancy, Nutrition and Infection (PIN) prospective cohort study to describe neighborhoods where low- and moderate-income pregnant women reside. Women who participated in the PIN study and who resided in Raleigh, NC and its surrounding suburbs were included (n = 703). Neighborhood attributes captured by the inventory included litter, housing condition, road condition, and social interactions that informed theoretical constructs of physical incivility, territoriality and social spaces. US Housing and Population Census 2000 data at the block group level were also assessed to identify the unique contribution of directly observed data. We hypothesize that neighborhood environments can influence health through psychosocial mediated pathways that lead to increased stress, or through disadvantage leading to poor neighborhood resources, or by protective attributes through increased social control.

**Results:**

Findings suggest that directly observed neighborhood attributes distinguished between different types of areas in which low-income pregnant non-Hispanic white and non-Hispanic black women lived. Theoretically informed scales of physical incivilities, territoriality and social spaces were constructed and found to be internally consistent. Scales were weakly associated indicating that these constructs capture distinct information about these neighborhoods. Physical incivilities, territoriality and social spaces scales were poorly explained by traditional census variables used to proxy neighborhood environment.

**Conclusion:**

If neighborhoods influence health through psychosocial mediated pathways then careful detailing of neighborhood attributes that contribute to stress or deterioration, beyond traditional socioeconomic status, are needed. We believe that measuring physical incivility, territoriality and social spaces as expressions of underlying issues of maintenance and social communication make important contributes to this field.

## Background

In the last two decades, research assessing neighborhood characteristics in the United States has expanded from exclusive reliance upon administrative records such as census data to directly observed measures. Census data, used as a proxy for neighborhood characteristics, have been critical for identifying important associations between socioeconomic disadvantage and a variety of adverse maternal and child outcomes such as maternal mortality [1], birthweight [2-11], preterm birth [12-15], neural tube defect [16], and infant mortality [1,17]. Associations between poor neighborhood socioeconomic environment, as measured by census data, and important health behaviors that may influence the course of pregnancy were also identified such as less physical activity [18], higher fat diets [19,20] and overweight among women but not men [21,22].

While census variables might approximate a neighborhood socioeconomic context, their utility is limited for several reasons. First, census data are available only at decennial intervals in the US, whereas neighborhood conditions can change within the span of a few years. Second, the exclusive use of census variables, which are produced by aggregating individual responses to census questions, implies that the important features of 'neighborhoods' can be captured by aggregating individual measures. This approach ignores the important role of contextual community features including the presence of facilities, the nature of social interactions, the quality of shared space, and the investments in infrastructure and community life that facilitate healthful activities, choices and interactions [23,24]. Third, while census variables continue to function as crude surrogates for neighborhood attributes, other aspects of the neighborhood need to be measured directly to more clearly understand pathways through which neighborhoods might influence health outcomes [25].

The shortcomings associated with census data have led to renewed appreciation of observational methods utilized outside the public health field and to the development of new tools designed to directly assess characteristics of the social and physical neighborhood environment [26-31]. Direct observation for data collection emerged largely from urban ecologic models that described the patterns and consequences of the growth and development of cities in the early part of the 20^th ^century [24,32,33]. Previous research suggests direct observation can produce reliable measures of neighborhoods and may offer specific insights into the neighborhood dynamics contributing to physical disorder, housing condition, territoriality expressions, social disorder, human interactions and evidence of alcohol, drug and tobacco use [34]. By selecting indicators of the probable mechanisms, directly observed data may more accurately define the populations at risk for adverse health outcomes and can identify the elements in this etiologic pathway that may be targeted by public policy interventions. Further, as the health impacts of neighborhood characteristics may vary by race and social class, we explicitly considered directly observed neighborhood attributes in the context of explaining racial or social class health disparities [19,35].

Three gaps in the literature were identified. First, direct observation of neighborhood attributes has mainly occurred in northern urban areas [26,28-31,36] and has yet to be conducted on urban areas of the new south; with the exception of New Orleans [27]. The new south is a term that describes the change in the US southern states from a largely agricultural to an urban/suburban region marked by social and economic changes, and rapid population growth due mainly to immigration of Hispanic and Asians to the region since the 1970s [37]. Second, research utilizing this approach, while generally collecting similar types of information (i.e., litter, broken windows), has not been standardized across localities, making comparison of the types of neighborhood attributes considered to influence health outcomes difficult [38]. Third, comparison of directly observed data to other, more standard neighborhood indicators, such as census data, has been limited.

We sought to address these research gaps by directly measuring neighborhood characteristics in Raleigh, NC and its surrounding suburbs for the Pregnancy, Infection and Nutrition study; a cohort study of risk factors for preterm birth. The purpose of this paper is to 1) describe the direct observation data collection effort conducted in urban and suburban areas representative of the new south; 2) describe neighborhoods and assess if neighborhood attributes differ by race; 3) compare prevalence of street segment level neighborhood attributes that comprise social and physical constructs between Raleigh, NC and Baltimore, MD where the survey was first created; and 4) assess the relationship between neighborhood characteristics and census variables traditionally used to characterize neighborhood socioeconomic conditions.

## Methods

### Study sample

Individual data and directly observable neighborhood attributes were collected as part of the Pregnancy, Infection, and Nutrition (PIN) cohort, a prospective study of determinants of preterm birth [39]. Participants were recruited from four prenatal care clinics in two settings: the University of North Carolina Residents' and Private Physicians' Obstetrics Clinics, the Wake County Department of Human Services, and Wake Area Health Education Center Prenatal Care Clinics. Between 1995 and 1999, 3,163 women were recruited into the study at 24 to 29 weeks' gestation, of whom, 973 reported their last address as within Wake County. Of these, 703 women whose addresses were within the city limits of Raleigh and its surrounding suburbs were included. Residential addresses were geo-coded by Geographic Data Technology (GDT), Inc., assigning latitude and longitude coordinates and census designations. Neighborhood-level data were collected on physical attributes such as housing condition, commercial property, and observable social interactions. Study procedures were in accord with the ethical standards of the Institutional Review Board of the University of North Carolina School of Medicine and Wake Medical Center.

## Data collection

### Individual Level

PIN participants completed a telephone interview at 26 to 31 weeks' gestation that solicited information on sociodemographic characteristics, health behaviors, psychosocial factors and previous as well as current medical history.

### Neighborhood instrument and protocol development and data collection

The Neighborhood Attributes Inventory was modified from a street survey developed in Baltimore, MD for a study that examined how neighborhood factors affected the cognitive and behavioral development of preschool age children [26]. The neighborhood attributes that were collected as part of this instrument were the indicators for social constructs related to the physical and neighborhood surroundings that might influence a stress response or behavioral change. We collected these neighborhood indicators because we believed these constructs were important contextual features for pregnant women as their presence might increase stress or influence poor health behaviors, such as decreasing physical activity, thereby affecting maternal health and fetal growth. PIN team researchers and maternal outreach veteran home visitors, who are lay health advisors that visit and assist pregnant women with prenatal care, reviewed the instrument. The instrument resulted in a 39-item survey representing four categories of neighborhood attributes: neighborhood physical conditions; social interactions; nonresidential land use (commercial property); and public, residential and nonresidential space (Additional file 1). The survey was pilot tested during five site visits. Ten students were hired and participated in a 30-hour training session that focused on inter-rater reliability; consistency of rating across time, space and person. Operational definitions for each item were established in the Neighborhood Data Collection Protocol. Inter-rater reliability tests were conducted twice during training and three times during data collection. Eighty-three percent agreement was achieved during training and maintained throughout data collection among pairs of raters.

PIN women were located in 115 of 263 (44%) Wake County block groups, which formed the sampling frame for street segment selection. Because of limited resources, a little over twenty percent of all street segments were randomly selected within the 115 block groups using Arcview ArcView 3.2a software (Arcview software, ESRI, 380 New York Street, Redlands, CA 92373-8100). PIN participants' street segments were added to the sample if they were not included among those randomly selected. A total of 2771 street segments comprised the final sample. Block groups were of variable size; the mean number of block group street segments was 24 (range, 6–66 street segments). Baltimore, MD, is distinct from Raleigh, NC in that it is a northeast urban area with jobs concentrated in the central city, has areas of concentrated poverty, and most neighborhood streets are laid out in a grid system. In contrast, Raleigh, NC, is more typical of the new south with a modest downtown containing government buildings, heavy suburban development, less concentrated population density and poverty and long, meandering streets. The average area of census block groups for Raleigh and its suburbs is 1.26 square miles (range, 0.10 to 15.64), considerably larger than the average area of 0.1 square miles (range, 0.02, 0.45) in Baltimore. In large part because of the non-grid street systems, opposing streets had inconsistent beginnings and endings. Therefore, street endings were defined as a natural break or intersection. The length of the street segments, the larger geographical area comprising a block group and the non-continuous nature of the street segments sampled within each block group necessitated a windshield audit, rather than a walking survey, to rate each street segment. The raters worked in pairs, driving each street segment up to three times between 9 am and 4 pm. Each street segment survey took 5–10 minutes to finish. Data collection was completed in 3 months during the summer of 2001.

## Measures

### Neighborhood definition

For this research, neighborhood was defined as the census block group because it represents the smallest census unit that may approximate one's neighborhood while still providing stable exposure estimates. Previous research in perinatal and children's health has found the block group to be an appropriate level of analysis for similar outcomes [3].

### Neighborhood scale development

Three theoretically informed scales were constructed based largely on previous research in Baltimore, MD: physical incivilities, territoriality and social spaces [26]. The first, signs of physical incivilities, a combination of physical disorder and poor housing condition, are theorized to communicate decreased local social control and may contribute to crime and further neighborhood deterioration [30]. Items comprising the physical incivilities scale included condition of housing, yards, commercial and public spaces, vacant or burned property, litter and graffiti. The second scale, territoriality, was comprised of indicators including fences, hedges, decorations, and signs, which serve as physical and symbolic demarcations of residential property, and are thought to communicate ownership and social control that lead to protective effects against crime and adverse community events [30,31,40]. The third scale, social spaces, was modified from the play spaces scale used by Caughy [26] to more fully capture the influence of diet, physical activity and stress on pregnancy. Eight variables were considered: presence of people, active people, non-resident visitors (police, service and delivery), yards, porches, parks, streets with low speed limits, sidewalks and racial diversity. Five items factored above 0.50 and were included in the social spaces scale: presence of people, non-resident visitors, parks, porches and sidewalks. Unrotated principle factor analysis of a correlation matrix among items was used to verify the underlying factor structure of the proposed latent variables and to obtain weights for each of the scale items. The three scales were constructed by summing the factor-weighted items.

### Census variables

Scales representing physical incivilities, territoriality and social spaces were then assessed for the extent of overlap with census variables traditionally used to estimate neighborhood level socioeconomic disadvantage, neighborhood stability and transportation. Sixteen 2000 US Census block group level variables were identified and assessed for their association with neighborhood scales. Census variables representing poverty (% below poverty, % public assistance, % female headed household with dependents), education (% no high school), employment (% unemployed), housing (median housing value, % with >1 person per room), occupation (% professional or management), racial composition (% white non-Hispanic, % Black non-Hispanic, % Hispanic), residential stability (% older than 65 years, % homes owned, % same residence since 1995), and transportation methods (% using private transportation to get to work, % using public transportation to get to work) were included.

### Statistical methods

Counts of each street segment neighborhood attribute were calculated, and a dichotomized indicator for presence/absence of each attribute was constructed. Block group proportions, the number of street segments with the attribute divided by the total number of segments rated, were calculated. In race-stratified analyses, proportions of block group attributes were compared using t-tests to explore how neighborhood attributes varied by race. Neighborhood scales were tested for internal reliability with Cronbach's alpha, and with maximum likelihood tests to assess two null hypotheses: that the number of true underlying factors is equal to zero, and that the number of true underlying factors is greater than one using a χ^2 ^test with p = <0.05. Spearman's correlation coefficient was used to assess association between the three scales and to assess the association between the scales and year 2000 census variables traditionally used to characterize neighborhood socioeconomic conditions, stability and transportation. An analysis of variance (ANOVA) was conducted to identify what proportion of the variance in the latent constructs, as represented by the physical incivilities, territoriality and social spaces scales, traditional socioeconomic census variables would explain. Analyses were conducted using Stata 8.2 [41].

## Results

### Description of PIN participants

Among the 703 Wake County PIN participants with complete address files, 27% were non-Hispanic white, 66% were non-Hispanic black and 7% were of other races/ethnicities. The mean age of PIN participants was 24 years (range, 16–40 years). Sixty-two percent were married, and 60% had a high school education or less. The mean income, as a percentage of the poverty level was 142% poverty (range, 8–857% poverty); 79% of the sample had incomes at or below 185% of the poverty level, the standard eligibility criteria for the Supplemental Food Program for Women, Infants and Children (WIC). As a whole, this sample could be characterized as a low- to middle-income population.

As a result of the economic and racial segregation of urban areas, we anticipated non-Hispanic white and non-Hispanic black women would live in qualitatively different neighborhoods in Raleigh, which was what we observed. Table 1 compares the mean values of selected neighborhood characteristics between non-Hispanic white and non-Hispanic black women. Every PIN woman was assigned the prevalence of each street level characteristic in her block-group as her neighborhood context value for that indicator. Then the mean value among non-Hispanic white women was compared to the mean value among non-Hispanic black women. There was a significant difference in mean values for most of the neighborhood attributes between these two groups of women. Non-Hispanic white women in this study were more likely to live in block groups that had a higher proportion of street segments with only single family dwellings (60.4 versus 50.0%) and with sidewalks (61.0 versus 49.6%), respectively; whereas non-Hispanic black women were more likely to live in block groups with litter (63.0 versus 41.4%) and no trespassing signs (21.8 versus 11.1%), respectively (Table 1). These differences persisted despite the PIN sample comprising mostly low-income women of both races.

**Table 1:** Selected neighborhood attributes, range, mean and standard deviation for total sample and by race

**Neighborhood Attribute**	**Range**	**Mean (n = 703)**	**Non-Hispanic white (n = 191)**	**Non-Hispanic black (n = 465)**
HOUSING & STREET ITEMS				

Presence of multiple dwellings	0–91%	32.2 (24.8)	27.7 (22.6)	41.8 (24.8)*
Presence of only single dwellings	9–100%	59.2 (24.0)	60.4 (19.8)	50.0 (23.4)*
Good housing condition	12–100%	81.5 (21.6)	85.3 (15.3)	73.1 (26.5)*
Presence of yards	57–100%	92.7 (13.3)	92.0 (11.7)	90.2 (11.7)
Good condition of yards	8–100%	74.4 (22.1)	77.4 (17.2)	68.0 (22.9)*
Presence of any litter	0–100%	48.1 (30.4)	41.4 (26.5)	63.0 (27.5)*
Presence of graffiti	0–17%	1.4 (0.3)	1.1 (1.9)	3.1 (4.5)*
Presence of sidewalks	0–100%	54.4 (26.0)	61.0 (23.4)	49.6 (19.9)*
Presence of street lamps	26–100%	89.0 (16.8)	80.6 (21.5)	91.2 (10.6)*

SOCIAL INTERACTION				

People present	0–70%	28.6 (16.8)	27.2 (12.5)	40.4 (19.1)*
Presence of parks	0–46%	6.3 (9.9)	4.3 (4.8)	6.8 (8.4)*
Presence of porches	9–90%	44.5 (20.0)	38.4 (15.0)	45.9 (20.2)*

SYMBOLIC AND PHYSICAL BOUNDARIES				

Presence of decorations	11–82%	57.6 (15.2)	59.9 (12.3)	51.2 (12.5)*
No Trespassing Sign	0–83%	13.0 (15.5)	11.1 (8.5)	21.8 (17.4)*
Neighborhood Sign	0–50%	11.8 (10.0)	14.1 (8.3)	15.7 (9.8)*
Community Watch Sign	0–57%	18.0 (13.8)	17.5 (11.7)	21.1 (11.6)*
Security Warning Signs	0–29%	10.5 (6.7)	9.6 (5.7)	13.5 (7.6)*
Presence of borders (hedges or fences)	0–71%	36.3 (12.8)	35.0 (12.6)	37.0 (12.3)

COMMERCIAL AND PUBLIC SPACES				

Presence of commercial buildings	0–94%	23.6 (20.8)	19.3 (15.2)	26.8 (17.3)*
Abandoned commercial building	0–50%	3.9 (9.4)	2.2 (8.0)	6.0 (11.0)*
Security bars on commercial buildings	0–100%	13.0 (20.7)	9.2 (15.0)	17.7 (18.6)*
Presence of new home construction	0–33%	1.8 (5.2)	2.9 (7.2)	1.9 (5.3)
Good condition of public spaces	12–100%	87.3 (14.0)	87.5 (11.0)	83.9 (17.7)*

* Probability of difference in scores (p = <0.05) using two sided t-test for mean differences compared to non-Hispanic white women

The neighborhood attribute data suggest that Raleigh NC, a city of the new urban south, may differ from the Baltimore, MD, our urban northeast example, in important ways. Items measuring physical incivilities, including graffiti, moderate/considerable litter, vacant/burned properties, poorly maintained yards, housing, and public spaces, were strikingly less prevalent in Raleigh than in Baltimore (e.g., 4% compared to 31% vacant residence, respectively) (Table 2). These findings suggest that there were fewer overt physical signs of incivilities in Raleigh, NC, or that incivilities might be manifested in other ways. Items measuring territoriality, including neighborhood watch, no trespassing and security warning signs, reaction of residents to raters, presence of borders and decorations, had similar prevalence rates for Raleigh and Baltimore. These findings suggest that residents of the new urban south and the urban northeast may mark their residential spaces similarly.

**Table 2:** Selected neighborhood attributes mean value at the street segment level for Baltimore, MD and Raleigh, NC

**Prevalence of physical incivility items among street segments in two cities**		
	Baltimore, MD (n = 1135)	Raleigh, NC (n = 2771)

Vacant residences	31.0	4.0
Poor ground condition	9.8	0.6
Moderate/considerable litter	25.0	4.5
Graffiti	39.0	1.4
Poor commercial building condition	11.0	1.8
Vacant commercial buildings	9.0	4.5
Poor condition of public spaces	33.0	1.8

**Prevalence of territoriality items among street segments in two cities**		

	Baltimore, MD (n = 1135)	Raleigh, NC (n = 2771)

Crime watch/security/no trespassing signs visible	73.7	65.6
Resident's reactions to raters	61.0	28.2
One third or more of homes with borders/hedges	41.0	58.5
One third or more of homes with security bars	25.2	Not present
One third or more of homes with decorations	61.0	67.6
Sign visible denoting neighborhood name	2.5	13.2

**Prevalence of play or social space items among street segments in two cities**		

	Baltimore, MD (n = 1135)	Raleigh, NC (n = 2771)

Presence of people	Not published	28.6
Children visibly playing	14.3	6.7
One third or more homes with yards	76.6	78.6
One third or more homes with porches	Not published	45.9
Nonresident visitors	Not published	18.0
Presence of parks	Not published	5.4
Parks in good condition	1.8	4.9
Street not a busy thoroughfare	70.8	77.7
Presence of sidewalks	Not published	44.4

The Cronbach's alpha coefficient for the physical incivility scale was 0.81, for social spaces was 0.61 and that for territoriality was 0.56 suggesting high and moderate internal reliability of the scales. The three scales appeared to represent unique latent constructs, as assessed by significant chi-square statistics (alpha = 0.05) which for each scale rejected both the null hypothesis that the number of true underlying factors is exactly zero, as well as the null hypothesis that the number of true underlying factors is greater than one. Therefore, we used the items that represented the scales previously published [26]. The scales were weakly correlated, the correlation between physical incivilities and territoriality was ρ = -0.05, between physical incivilities and social spaces was ρ = 0.39, and between territoriality and social spaces was ρ = -0.03, indicating the scales represent distinct latent constructs.

### Association of scales with 2000 US Census variables

Presented in Table 3 are the Spearman's correlation coefficients between the scales for physical incivilities, territoriality and social spaces, and 16 block group level census variables. Correlations between the physical incivilities scale and census variables ranged between 0.16 (% same residence since 1995) and 0.68 (% no high school education). Generally, the physical incivilities scale was moderately and positively associated with non-Hispanic black race, poverty, and low education, and negatively associated with non-Hispanic white race, professional occupation and housing value (ρ ≥ 0.5). Census variables representing proportion elderly and Hispanic residents, employment status, housing, residential stability and transportation were not highly correlated with physical incivilities. We did not anticipate high or moderate correlations (ρ ≥ 0.5) between socioeconomic census variables and territoriality. There were weak correlations between the territoriality scale and socioeconomic census variables ranging from 0.00 (% female headed households with dependents) to 0.22 (% below poverty), moderate correlations with census variables that are used to represent residential stability, from 0.45 (% older than 65 years) to 0.58 (% same residence since 1995), and weak correlations with transportation variables. Lastly, we correlated census variables with social spaces and hypothesized that few would be associated with social spaces above ρ = 0.50, and that census measures of public and private transportation use might have higher association with social spaces than census variables used to capture socioeconomic status or residential stability. Correlations between social spaces and socioeconomic variables ranged from 0.05 (% Hispanic) to 0.43 (% poverty), from 0.01 (% older than 65 years) to -0.43 (% homes owned) for residential stability, and were moderately correlated with transportation.

**Table 3:** Spearman's correlation coefficient among three scales and 16 census variables at the block group level

**2000 Census Block Group Variables (n = 115)**	**Physical Incivility**	**Territoriality**	**Social Spaces**
POVERTY			
% Income Below Poverty	0.62*	-0.22*	0.43*
% Public Assistance	0.47*	0.22*	0.14
% Female Head of Household with Dependents	0.44*	0.00	0.13
EDUCATION			
% No High School Diploma	0.68*	0.04	0.21*
EMPLOYMENT/OCCUPATION			
% Unemployed	0.43*	-0.10	0.09
% Occupation is Management or Professional	-0.62*	-0.10	-0.12
HOUSING			
Owner Occupied Median Housing Value	-0.56*	-0.02	-0.06
% ≥ 1 Person per Room (crowding)	0.33*	-0.09	0.10
RACIAL COMPOSITION			
% Black non-Hispanic	0.63*	0.11	0.14
% White non-Hispanic	-0.58*	-0.06	0.12
% Hispanic	0.17	-0.12	0.05
RESIDENTIAL STABILITY			
% Older than 65 years	-0.26*	0.45*	0.01
% Homes Owned	-0.43*	0.47*	-0.43*
% Living in Same Residence since 1995	-0.16	0.58*	-0.20*
TRANSPORTATION			
% Using private transportation to get to work	-0.38*	0.03	-0.56*
% Using public transportation to get to work	0.43*	0.05	0.36*

* Significant at ρ < 0.05

Variance in the scales explained by traditional census variables used to capture neighborhood disadvantage, residential stability and transportation was assessed using ANOVA [2,3,5,12]. First, the proportion of variance in physical incivility explained by poverty alone, the most commonly used census variable to account for neighborhood disadvantage was 56%. Census variables correlated above 0.5 with physical incivilities were then assessed. Adding to census tract poverty was % no high school, median housing value, % professional or management occupation, and % non-Hispanic black which together explained 62% of the variance in the physical incivilities construct. Three census variables modestly correlated at 0.4 or greater with the territoriality scale – % same residence since 1995, % older than 65 years and % of homes owned – were used to assess and only explained 40% of the variance in the territoriality construct. Three census variables modestly correlated at 0.4 or greater with the social spaces scale – % poverty, % of homes owned, and % private transportation to get to work – were used to assess and only explained 41% of the variance in the social spaces construct. The finding of moderate to high internal reliability based on the Cronbach's alpha and that census variables capture 62%, 40% and 41% of the physical incivilities, territoriality and social spaces scales, respectively, suggest the scales depict unique information about these neighborhoods, not obtainable using traditional census measures.

## Discussion

This research sought to describe the neighborhood environment of Raleigh, NC, a city of the new urban south, as part of a cohort study of risk factors for adverse pregnancy outcomes. The new south is rapidly growing and may experience neighborhood changes in resources and maintenance that may be important to capture through direct observation. Conducting a windshield tour of Raleigh, NC and surrounding suburbs was necessary because of the large geography and low population density. Although direct observation data were collected via driving, we found we were able to use a data collection instrument previously used in Baltimore, MD to capture neighborhood attributes.

The second objective of this paper was to analyze race-stratified neighborhood attributes, indicating that, within the PIN sample, low-income non-Hispanic white and non-Hispanic black women live in qualitatively distinct neighborhoods. We found that non-Hispanic white women lived in neighborhoods with more amenities such as sidewalks, whereas non-Hispanic black women lived in neighborhoods characterized by more markers of incivilities. Based on theories of psychosocial etiology for adverse reproductive outcomes [42,43], these very different environments may have important effects on racial disparities in preterm birth, a profound health disparity in the US, especially in the US south.

This particular neighborhood observation tool was chosen because the three theoretically informed constructs of physical incivilities, territoriality and social spaces are hypothesized to influence intermediate health outcomes during pregnancy such as stress level, diet, physical activity and weight status, as well as delivery outcomes of birthweight and preterm birth. Physical incivilities, characterized by poor housing, litter and abandoned houses, may directly and indirectly influence stress by increasing allostatic load or by influencing behaviors that help maintain low stress levels. Feelings of being unsafe might influence psychologically mediated pathways increasing stress and a physiological response to stress that over time increases a woman's allostatic load [44]. This chronic stress condition has been presented as a weathering effect that over time influences poor health outcomes [45]. Signs of physical incivilities that increased stress and decrease perceived safety may influence behavioral changes [46] such as the inability to exercise in one's own neighborhood [18] or increased gonorrhea rates [27]. Conversely, territoriality is thought to communicate social control and have a protective affect on health, perhaps lowering allostatic load or increasing confidence to walk within one's neighborhood. The social spaces construct is hypothesized to promote personal interaction thereby increasing opportunities for social control and activity within one's neighborhood. To the extent that stress mediated pathways are involved in health outcomes, this neighborhood survey may be applicable for the study of other health outcomes such as weight status or chronic diseases.

Our research also sought to compare the attributes of a Raleigh, NC and its suburbs, a city of the new urban south, with those of Baltimore MD, a city with characteristics of the northern urban industrial center. Contrasting neighborhood attributes from various geographies is important because regardless of different developmental histories, similarities in neighborhood physical and observable manifestations that persist may help us understand how neighborhoods are important to health [38]. Despite the scarcity of items representing incivilities in the Raleigh area, the physical incivilities scales had high internally reliability based on Cronbach's alpha scores, and territoriality and social spaces had moderate internal reliability. The low correlation estimates among the scales suggested that the scales captured distinct constructs and provided unique information about neighborhood attributes. We hypothesize that physical incivilities, territoriality and social spaces are importantly associated with reproductive health outcomes in Raleigh, NC and its surrounding suburbs, largely through psychosocially mediated pathways [42].

The fourth objective of this paper was to demonstrate that the unique neighborhood information obtained through direct observation is distinct from that of traditionally used census data. While the markers for incivilities, territoriality and social spaces may be used to estimate neighborhood deterioration, upkeep or resident investment, census variables can not replicate the information provided by these scales. Further, the theoretically informed scales suggest a mechanism regarding how neighborhoods can influence health outcomes. The inadequacy of using poverty as a surrogate for neighborhood dynamics is due to heterogeneity across low-income neighborhoods with regards to disadvantage, crime, and resources, as has been observed in previous studies [47]. In a study of neighborhood effects on gonorrhea rates in New Orleans, LA, Cohen et al. found that a "Broken Windows" index – a directly observed measure combining housing condition, graffiti, accumulated garbage, abandoned vehicles and public high schools with problems – distinguished among low-income neighborhoods [27]. Low-income, low broken windows indexed neighborhoods had significantly lower gonorrhea rates than low-income, high broken windows indexed neighborhoods. These illustrations show the importance of using directly observed data in combination with census or other administrative data; geo-referenced data such as parks, commerce, schools, zoning, alcohol outlets, and crime data [27,47]; and perceived neighborhood environment data [48], to provide a rich picture of neighborhoods and their attributes, with minimal investment of time and expense, and to better understand mechanisms of neighborhood influences on health. In addition, increased accessibility to geocoded data has enabled more sophisticated modeling techniques and permit exposures to be characterized as simple counts or as rates for various units of geographic analysis [49,50]. Geocoding allows one to observe the spatial distribution of an exposure over multiple geographies to identify hot spots, assess spatial autocorrelation, and allows the creation of accessibility measures and geo-simulation [51]. The utility of different modeling techniques permits exploration of the most relevant exposure classification for health outcomes. In this way not only can the relationship of geography be better understood but the influence of changes in terrain on health can be assessed enabling researcher to explore causal mechanisms and move beyond simple associations.

Although newly developed southern US cities are notably less segregated than the industrial centers of the northeast [52], and patterns of poverty and neighborhood development are different because of the growth of these areas in an era since the demise of heavy industry as the basis for economic organization [53], the recent establishment of these communities may provide fewer social resources that could help to buffer effects of harmful environments. Furthermore, cities in which major growth has occurred since the automobile became ubiquitous are more geographically dispersed and may reduce easy access to facilities and amenities compared to cities with concentrated population centers and long-established urban transit systems. Reduced service concentration may be especially burdensome for poor individuals and families who may not own a car or have hours to devote to traveling between service facilities. Furthermore, recent growth in new south centers such as Raleigh, Charlotte and Atlanta has occurred since the era of suburban flight, meaning that center-city areas were never abandoned, since the center city never gained prominence in this later era. This implies a lower prevalence of the 'incivilities' that emerge when populations abandon decaying areas of the city for opportunities in newer suburbs. Yet, even with the lower prevalence of incivilities, their existence may influence health outcomes, and as population growth and development occurs, incivilities in poorly maintained neighborhoods may increase.

Future research is needed to corroborate data collection methods and findings. Directly observed neighborhood attributes can be combined with geographic information systems and resource inventories to validate findings, and can be augmented by these sources and census data to provide a detailed contextual database for the analysis of neighborhoods' influences on health outcomes. Longitudinal data collection and analysis of individuals and the neighborhoods in which they reside will be important as we move forward with this research. Analysis using the physical incivilities, territoriality and social spaces scales to predict health outcomes, particularly adverse birth outcomes is needed and forthcoming.

## Authors' contributions

BAL was involved in the study design, implementation, data analysis, execution and writing up of the draft and final copies of the manuscript. LM was involved with the implementation, data entry, data analysis, and preparation of the manuscript. JSK was involved in the study design, interpretation of results and preparation of the manuscript. ND conceived the need for the study, was involved in the study design and preparation of the manuscript. MC assisted with the interpretation of the findings and was involved with the preparation of the manuscript. POC served as a consultant for the project, was involved with the interpretation of the findings and preparation of the manuscript. DAS conceived the need for the study, was involved in the study design and preparation of the manuscript.

## Supplementary Material

Additional File 1 The 39-item neighborhood survey instrument in pdf format. Click here for file
