# Supplementary material for: Direct observation of neighborhood attributes in an urban area of the US south: characterizing the social context of pregnancy
Source: Int J Health Geogr. 2006 Mar 17;5:11. doi: 10.1186/1476-072X-5-11 (PMC1444926; doi:10.1186/1476-072X-5-11)
Supplement: Additional File 1 — The 39-item neighborhood survey instrument in pdf format. [file 1476-072X-5-11-S1.pdf]

Block group + street segment ID :______________

PATID ______________________ ____________
Block Group No. ____________________________
Street Address ______________________________
Date: ________________Rater:_________________
Start time: ____________End time: ______________

Neighborhood Physical Conditions

1. Types of residential housing (Code all that apply)
   None **(Skip to # 15)** ………………………. 0
   Single family detached dwellings…………..1
   Trailer home/mobile home………………… 2
   Multiple occupancy (2-6 units)……………. 3
   Apartment building (7 or more units)….….. 4
   Housing authority projects………...…….. 5
   New construction …………………………. 6
   Renovation ……………………………….. 7

2. Main type of residential housing (Code more than one if necessary)
   Single family detached dwellings……….. 1
   Trailer home/mobile home …………………2
   Multiple occupancy (2-6 units)……………..3
   Apartment building (7 or more units)… ….. 4
   Housing authority projects ……………..….. 5
   New construction …………………………. 6
   Renovation ……………………………….. 7

3. Overall condition of most residential units
   Excellent ………………………………….. 1
   Good condition/well kept …………………. 2
   Fair condition ………………………………3
   Poor/deteriorated condition ……………….. 4
   Mixed condition ……………………………5

4. Overall condition of resident-kept grounds
   Not applicable …………………………… 0
   Excellent ………………………………… 1
   Good condition/well kept ………………… 2
   Fair condition ………………………………3
   Poor/deteriorated condition ……………… 4
   Mixed conditions (extreme differences) … 5

5. Indication of neighborhood/block uniformity
   No ………………………………………… 0
   Yes …………………………………………1

6. Number of residential housing units _______

7. Number of For Sale or For Rent Signs _______

8. Number with visible security warning signs _______

9. Number of burned, boarded up or abandoned _______

10. Number with front yard _______

11. Number with porches _______

12. Number with some form of decoration _______

13. Number with border (fences/shrubs) _________
    (If 0, skip to # 15)

14. Average height of borders (fences/shrubs)
    Below shoulder level ……………………… 1
    Above shoulder level ……………………… 2
    Mixed ……………………………………. 3

Social Interactions

15. People **(If 0, skip to # 22)**
    Number of visible people (adults + children) ______

16. People from more than one racial group
    No ………………………………………… 0
    Yes …………………………………………1

17. Adult activity (Code all that apply)
    If no adults present ………………………. 0
    Walking …………………………………… 1
    Socializing (Talking with neighbors) ………2
    Socializing in mixed racial groups …………3
    Home repair, landscaping, or car care …….. 4
    Sitting/standing on porch or stoop …………5
    Supervising children …………………….. 6
    Patronizing business establishments ……… 7
    Standing on the sidewalk ………………… 8
    Sitting/standing at the bus stop …………… 9
    Getting into or out of vehicles …………… 10
    Walking a dog ……………………………11
    Conducting home-based vending …………12
    Recreational activity (e.g., jogging) ………13
    Other _________________________ …… 14

18. Reaction of people to raters (Code all that apply)
    None or very little notice taken …………… 0
    Glances curiosity, wave or hello……………1
    Blatant speculative stares …………………. 2
    Residents ask raters what they are doing …. 3
    People telling raters what to do for neighborhood……………………………… 4

19. Children under 12 years old
    Number of children …………………..______
    (If 0, skip to # 22)

20. Proportion of children under adult supervision
    None ……………………………………… 0
    Less than one third …………………………1
    One third to one half …………………….. 2
    More than half …………………………… 3

21. Children's activities (Code all that apply)
    Sitting or standing on porch or front yard…..1
    Playing on the porch or front yard …………2
    Sitting, standing or playing in the street …... 3
    Sitting standing or playing in a vacant lot … 4
    Walking on the sidewalk or street ……..….. 5
    Playing at park or play ground ……………. 6

Block Group No. ______________________
Street Address _________________________

22. Park or playground
No **(Skip to # 25)**………………………….. 0
Yes (public) ……………………………….. 1
Yes (private) ………………………………..2

23. Condition of playground/park
Good condition/well kept …………………..1
Fair condition ………………………………2
Poor/deteriorated condition ……………….. 3

24. Occupants of the playground/park
None ………………………………………..0
Adults only …………………………………1
Adults with children ………………………..2
Teen groups ………………………………...3
Groups of unaccompanied children ……….. 4
Mixed usage ………………………………..5

25. Nonresidential visitors (Code all that apply)
There are none …………………………….. 0
Mormons or other religious based visits ….. 1
Mobile vendors/food peddlers……………. 2
Civic/social service agents …………………3
Police officers on foot/vehicle/horse……… 4
Utility/repair/delivery people ………………5
People passing through …………………… 6
Others ________________________ ……… 7

Nonresidential Land Use

26. Presence of nonresidential land use (commercial)
No **(Skip to # 30)** ………………………….. 0
Yes (Code all that apply)………………… 1
New construction ………………………… 2
Renovation ………..…………………… 3

27. Overall condition of most of the buildings
Excellent ………………………………… 1
Good condition/well kept ………………… 2
Fair condition …………………………….. 3
Poor/deteriorated condition ……………… 4
Mixed conditions (extreme differences) … 5

28. Proportion with security bars/gratings
None ……………………………………… 0
Less than 1/3 ……………………………… 1
One third to one half ……………………… 2
More than half …………………………… 3

29. Proportion of burned, boarded up or abandoned
None ……………………………………… 0
Less than 1/3 ……………………………… 1
One third to one half ……………………… 2
More than half …………………………… 3

Public, residential and non residential space

30. Proportion of the land that is vacant / underdeveloped
None **(Skip to #32)** ……………………….. 0
Less than 1/3 …………………………….… 1
One third to one half ……………………… 2
More than half …………………………… 3

31. Overall condition of underdeveloped property
Good …………………………………… 1
Fair …………………………………………2
Poor (illegal dumping) …………………… 3

32. Amount of litter
None **(Skip to # 34)**………………………. 0
A little ……………………………………. 1
A moderate amount ………………………. 2
A considerable amount …………………… 3

33. Type of litter (Code all that apply)
Paper, cans/bottles nonalcoholic ………….. 1
Can/bottles alcoholic ……………………….2
Drug paraphernalia …………………………3
Clothing items …………………………….. 4
Furniture ……………………………………5
Tires ……………………………………….. 6
Appliances (small) ………………………… 7
Appliances (large) ………………………… 8
Abandoned vehicles ………………………. 9
Other:______________________________10

34. Amount of graffiti
None ……………………………………… 0
A little …………………………………… 1
A moderate amount ……………………… 2

35. Type of street segment
Major thoroughfare/busy street …………… 1
Moderately busy thoroughfare ……………. 2
Side street ………………………………… 3
Dead-end street …………………………… 4
One way street …………………………….. 5
Cul-de-sac street ………………………… 6

36. Presence of sidewalks and street lamps
(Code all that apply)
Neither …………………………………… 0
Sidewalks …………………………………. 1
Street lamps ………………………………. 2

37. General condition of public spaces
Good ……………………………………… 1
Fair …………………………………………2
Poor ……………………………………… 3
Mixed conditions ………………………… 4

Block Group No. ______________________
Street Address ________________________

38. Visible Signs (Code all that apply)

None ……………………………………….	0
Neighborhood sign ………………………..	1
Community watch sign …………………….	2
No trespassing sign or no solicitation ……..	3
Public transportation stops …………………	4
Commercial advertising/ Help wanted……	5
Political events/campaigns ………………..	6
Neighborhood, social, cultural events ……	7
Drug Free Zone …………………………...	8
Home-based business ……………………..	9
No Dumping ………………………………	10
Other __________________________……	11

39. Parking or traffic signs (Code all that apply)

None ………………………………………	0
Speed limit …………………………………	1
Stop sign/stop light…………………………	2
Residential parking ………………………...	3
No parking/stopping ………………………	4
School zone ………………………………..	5
Children playing …………………………..	6
Street crossing button/crosswalk …………	7
Other ________________________ ………	8

Nonresidential Land Use Tally Sheet

❑ BUSINESS / INDUSTRY
1. _________ [ ] Manufacturing light
2. _________ [ ] Manufacturing heavy
3. _________ [ ] Professional offices
4. _________ [ ] Warehouse

❑ COUNTY/ PUBLIC SERVICES / UTILITIES
5. _________ [ ] Criminal justice facilities
6. _________ [ ] Employment offices
7. _________ [ ] Fire station
8. _________ [ ] Parking lot
9. _________ [ ] Police Station
10. ________ [ ] Political/community/civic
11. ________ [ ] Social service, welfare offices
12. ________ [ ] Utility company (e.g., gas)

❑ FOOD
13. ________ [ ] Convenience stores
14. ________ [ ] Fast food/take out places
15. ________ [ ] Restaurants
16. ________ [ ] Supermarket/grocery stores

❑ HEALTH CARE
17. ________ [ ] Drug stores/pharmacies
18. ________ [ ] Health clinic
19. ________ [ ] Hospitals

❑ PRIVATE SERVICES
20 ________ [ ] Automobile repair/body shop
21. ________ [ ] Banks
22 ________ [ ] Barber shop or beauty salon
23. ________ [ ] Business services-printing/copying
24. ________ [ ] Check cashing service
25. ________ [ ] Dry cleaning/tailoring/laundromat
26. ________ [ ] Gasoline station
27. ________ [ ] Real Estate office
28. ________ [ ] Travel agents

COMMENTS:_________________________________
____
____

❑ RECREATION
29. ________ [ ] Bars
30. ________ [ ] Movie Theater
31. ________ [ ] Recreational service
32. ________ [ ] Sex entertainment shops
33. ________ [ ] Video games/pool/bowling

❑ RELIGIOUS
34. ________ [ ] Cemetery
35. ________ [ ] Church/religious center
36. ________ [ ] Churches/storefront
37. ________ [ ] Funeral home

❑ USED GOODS SALES
38. ________ [ ] Automobile sales/rental used
39. ________ [ ] Furniture store used
40. ________ [ ] Pawn Shop
41. ________ [ ] Second hand stores

❑ RETAIL / SALES
42. ________ [ ] Appliance sales, rental, repair
43 ________ [ ] Automobile sales/rental new
44. ________ [ ] Clothing stores
45. ________ [ ] Electronic stores
46. ________ [ ] Furniture stores new
47. ________ [ ] Home repair/hardware/lumber
48. ________ [ ] Liquor stores
49. ________ [ ] Specialty retailers
50. ________ [ ] Variety stores

❑ SCHOOLS /CHILDCARE
51. ________ [ ] Day care centers
52. ________ [ ] Public school (primary-secondary)
53. ________ [ ] Technical school
54. ________ [ ] Schools-Colleges
55. ________ [ ] Private parochial school
56. ________ [ ] Private non-parochial school

❑ 57. OTHER_________________________________
____
____
